# Iris Color and Lens Thickness in Chinese Teenagers

**DOI:** 10.1167/tvst.7.5.25

**Published:** 2018-10-10

**Authors:** Chen-Wei Pan, Ou Zhang, Dan-Ning Hu, Rong-Kun Wu, Jun Li, Hua Zhong, Min Hu

**Affiliations:** 1 School of Public Health, Medical College of Soochow University, Suzhou, China; 2 Department of General Medicine, Kunming Children's Hospital, Kunming, China; 3 New York Eye and Ear Infirmary at Mount Sinai, Icahn School of Medicine at Mount Sinai, New York, NY, USA; 4 Department of Ophthalmology, the Second People's Hospital of Yunnan Province, Kunming, China; 5 Department of Ophthalmology, the First Affiliated Hospital of Kunming Medical University, Kunming, China

**Keywords:** iris color, lens thickness, epidemiology

## Abstract

**Purpose:**

We aimed to determine the association of iris color with lens thickness (LT) in a school-based sample of Chinese teenagers.

**Methods:**

In total, 2346 grade 7 students, from 10 middle schools, aged 13 to 14 years in Mojiang located in Southwestern China were included in the analysis. A grading system was developed to assess iris color based on standardized slit-lamp photographs. LT was measured by the LenStar LS900. Refractive error was measured after cycloplegia using an autorefractor and ocular biometric parameters, including axial length (AL), were measured using an IOL Master.

**Results:**

There was a significant trend of decreasing LTs with darker iris color. On average, eyes with “grade 1” (the lightest) iris color, when compared with those with “grade 5” (the darkest), had greater LTs (mean difference, 0.1 mm). After adjusting for other potential confounders including sex, height, and ALs in generalized estimating equation models, the trend was similar and did not change significantly. Compared with individuals with iris color of grade 1, those with grade 5 had a thinner lens of 0.1 mm (95% confidence interval [CI]: 0.01, 0.19) in sex-adjusted model and a 0.09 mm (95% CI: 0, 0.18) in multivariate-adjusted model.

**Conclusions:**

Lighter iris color might be associated with greater LTs in Chinese teenagers. The biological mechanisms underlying the association warrant further clarification.

**Translational Relevance:**

As LT is an important refractive component, knowledge on the effect of iris color on LTs may assist in the design of novel technologies, which could control refractive development.

## Introduction

Iris color, one of the most obvious physical characteristics of human beings, fully develops during infancy and does not change significantly in adulthood.^1^ Epidemiologic studies have revealed that iris color may have important implications regarding the clinical management of several eye diseases. There have been sufficient evidence linking iris color with a series of ocular conditions including age-related cataract,^2–4^ age-related macular degeneration,^5,6^ and uveal melanoma.^7^ In our previous work, we found that adolescents with darker iris color tended to have more myopic refractive errors and longer axial lengths (ALs).^8^ However, the biologic mechanisms underlying these observed associations between iris color and ocular conditions remain unclear.

Understanding the inter-relationship between iris color and ocular biometric components may help to shed some light on the biologic mechanisms of ocular conditions associated with iris color. There have been limited data assessing the relationship between iris color and ocular biometric components. Irides with different colors are supposed to have different filtering rates for ultraviolet (UV) radiation and visible lights with different wavelengths.^9^ Crystalline lens is an important component of optical system of the eye. The amounts of UV and visible light pass through the iris and enter the nonpupillary area of the lens are determined by iris color. We hypothesize that the growth of crystalline lens thickness (LT) might be influenced by the amounts of UV and visible lights passing through the iris. Therefore, variations in LTs may be associated with irides with different colors. In this study, we examined the relationship between iris color and the variations in LTs in a school-based sample of Chinese students. The results would have clinical implications regarding the potential impacts of lights on ocular characteristics.

## Methods

### Study Population

The data of this study were obtained from the baseline examinations of the Mojiang Myopia Progression Study, which is a school-based cohort study on the prevalence, incidence, and predictors of myopia in school students in rural China. The baseline examinations of this study were conducted in 2016. Detailed study protocols and some other major findings have been reported elsewhere.^8,10,11^ In brief, the original study cohorts included elementary school grade 1 students and middle school grade 7 students in Mojiang, which is located in Yunnan Province in the Southwestern part of China. The analysis in this paper focused on grade 7 students as data on iris color were only measured in this cohort. A total of 2346 grade 7 students participated in the baseline survey with complete data obtained and the response rate of this cohort was 93.5%. There were no sex differences between participants and nonparticipants (*P* = 0.25).

The Mojiang Myopia Progression Study was performed in accordance with the tenets of the Declaration of Helsinki. The study protocol was approved by the institutional review board of Kunming Medical University. Written informed consent was obtained from at least one parent or legal guardian of each participant.

### Measurement of LT

In this study, LT was measured by the LenStar LS900 (Haag-Streit, Bern, Switzerland). We followed the standardized protocols as recommended by the manufacturer. Participants were seated and their heads were stabilized using a chin rest and brow bar when measurements were performed. Study optometrists aligned the instrument using the image of the eye on the computer monitor. Participants were asked to blink just before measurements being taken and fixate on the internal fixation light during the measurements. The instrument automatically detected blinking or loss of fixation and measurements were repeated in this case. Five readings were taken for each eye and the mean of them was used in subsequent analyses.

### Iris Color Grading

The detailed grading protocol of iris color has been described in our previous report on the association between iris color and refractive error.^8^ In brief, we obtained standardized slit-lamp photographs of anterior segment of the eye and developed a grading system assessing iris color. A panel of reference photographs was selected, which best representing the variations in iris color observed in the sample (shown in a previous report).^8^ Two graders with reasonable intragraders agreement independently graded the color of all the iris photographs by comparing the specific photograph with the reference panel. The kappa index of the two graders was 0.74 in a pilot test. In addition, one grader repeated the grading of 50 photographs after 2 weeks to assess the intrarater agreement, which was 0.88 in this study. “Grade 1” denoted the lightest color while “grade 5” denoted the darkest. The higher grade was assigned if a photo was considered to be between two consecutive grades. Inconsistencies between the two graders were solved by the third grader.

### Measurement of Other Covariates

Refractive error was measured after cycloplegia using an autorefractor (RM-8000; Topcon Corp., Tokyo, Japan). Ocular biometric parameters such as AL, anterior chamber depth (ACD), and corneal power (CP) were measured using an IOL Master (Carl Zeiss Meditec AG, Jena, Germany). Blood pressure was measured according to the protocol as recommended by the National High Blood Pressure Education Program Working Group on children and adolescents.^12^ To measure blood pressure in a controlled environment, participants sat in a chair for 5 minutes of rest, while the right arm was supported at the heart level. Blood pressure was measured with a mercury column sphygmomanometer and then the first and fifth Korotkoff sounds were used to determine the systolic and diastolic blood pressure. Height was measured to the nearest 0.1 cm by a wall-mounted measuring tape. Participants stood straight, barefoot, with relaxed shoulders and their arms hanging freely. Weight was measured to the nearest 0.1 kg by a scale, with minimal clothing and without shoes. Waist circumference was measured to the nearest 0.1 cm by an inelastic measuring tape midway between the lowest rib and the superior border of the iliac crest at the end of a normal exhalation. All the anthropometric indices were measured twice and the mean value was recorded. Body mass index (BMI) was calculated as the weight in kilograms divided by the square of the height in meters.

### Statistical Analysis

Data analysis was performed using SPSS version 18.0 (Statistical Package for Social Science; SPSS Inc., Chicago, IL). The distribution of LTs was compared across different grades of iris color in univariate analysis. Generalized estimating equation (GEE) models with the right and left eye data combined were fitted to estimate the associations between iris color and LTs. We only adjusted for sex in the first model. In the second model, we additionally adjusted for covariates were significantly different in univariate comparison (*P* < 0.10). Subgroup analyses were performed to examine whether the findings were consistent across categories of possible confounders.

## Results

**Figure 1:**
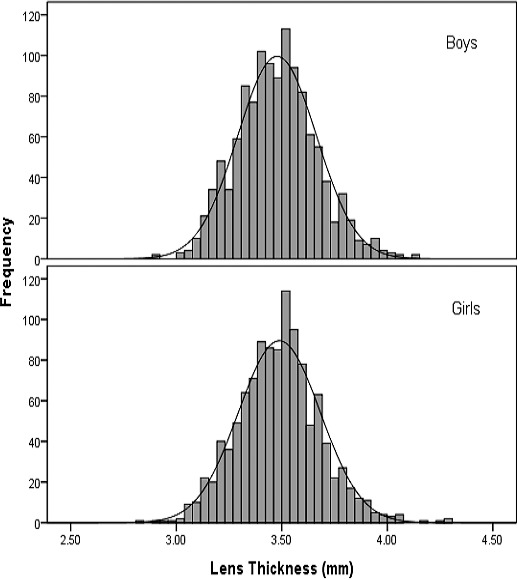
Frequency distributions of lens thickness in boys and girls.

The correlation of LTs between the left and right eyes was high (correlation coefficient = 0.94, *P* < 0.001). The mean LT for the overall population was 3.48 (standard deviation [SD]: 0.19) mm with no significant sex differences observed (*P* = 0.57). The distribution of LTs in boys and girls is shown in Figure 1. LTs for the overall population did not demonstrate normal distribution (Kurtosis = 0.43, Skewness = 0.26, *P* for K-S test = 0.02). When stratified by sex, LTs were normally distributed. Table 1 compares the characteristics of participants with different levels of LTs as stratified by quartiles. In general, there were no significant trend of LTs with height, weight, BMI, waist circumference, systolic, and diastolic blood pressure (all *P* > 0.10). LT was negatively related with AL, ACD, and CP (all *P* < 0.001). In addition, more myopic refractive error was associated with decreased LT (*P* < 0.001).

**Table 1:**
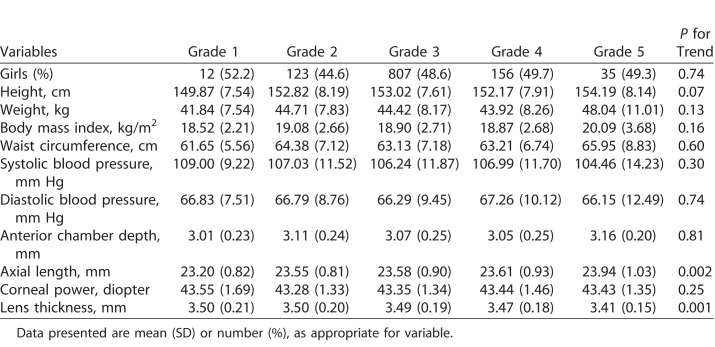
Characteristics of Participants With Different Grades of Iris Color

**Figure 2:**
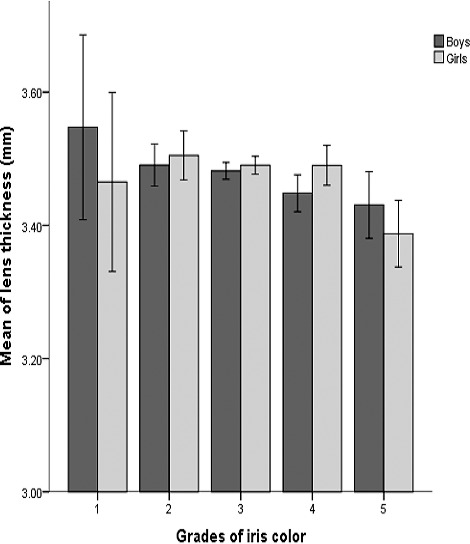
Mean lens thickness by different grades of iris color; error bars correspond to 95% confidence intervals.

Figure 2 demonstrates the mean LTs stratified by the grades of iris color in boys and girls. There was a significant trend of decreasing LT with darker iris color. On average, eyes with “grade 1” iris color, when compared with those with “grade 5”, had greater LTs (mean difference, 0.1 mm).

GEE models were constructed to evaluate the independent effect of iris color on LT. In the first model using “grade 5” as the reference group, we only adjusted for sex and found that lighter iris color was associated with increasing LTs (*P* for trend < 0.001). After additionally adjusting for other potential confounders including height and ALs, the trend was similar and did not change significantly. For example, compared with students with iris color of grade 1 (the lightest), those with grade 5 (the darkest) had a thinner lens of 0.1 mm (95% confidence interval [CI]: 0.01, 0.19) in sex-adjusted model and a 0.09 mm (95% CI: 0, 0.18) in multivariate adjusted model (Table 2).

**Table 2:**
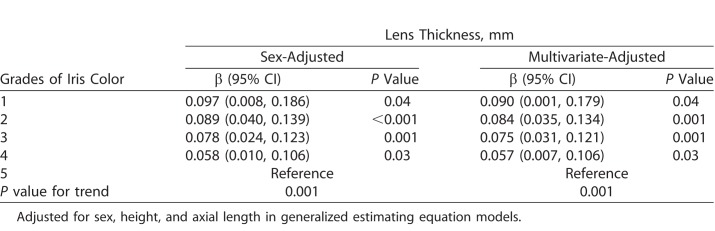
Associations of Grades of Iris Color With Lens Thickness

## Discussion

In this study, we observed an association of iris color with LTs among year 7 school students in rural China. After controlling for the effect of sex and potential confounders, students with lighter iris color tended to have thicker lens and dose-response relationships were observed.

There are two different pigment cells in the iris, the iris pigment epithelial cells and iridial melanocytes. The pigment epithelial cells are located at the posterior surface of the iris and the melanocytes are located in the iris stroma and in the anterior surface of the iris.^13^ Iris pigment epithelial cells are pigmented in all races and colors. The quantity and type of melanin in the iris melanocytes vary with iris color.^13^ Therefore variation of iris color is determined by the variation in pigmentation of the iris melanocytes, not by the iris pigment epithelial cells under physiological circumstance.^13^

There are three different hypothesis on the relationship between the lens thickness and the color of the iris. First, the increase of the lens thickness causes a slight bulge forward of the anterior surface of the lens, which results in an slight bulge forward of the iris that attached on the anterior surface of the lens. The posterior end of the iris (the iris root) is fixed at the anterior surface of the ciliary body, therefore, the move forward of the iris may cause a slight elongation and increase of the iris area. The color of any pigmented tissue (including the iris) is mainly determined by melanin content per area.^14^ Therefore, the increase of the lens thickness may cause a slight dilution of melanin in the iris and results in a slight decrease of iris pigmentation. Second, UV radiation at a high dose can cause apoptosis of lens cells and results in cataract formation.^15^ However, UV radiation at small dosage may also stimulate the development of the lens. It has been reported that Wnt-4 is expressed in developing eye and is required for the development of the eye (including the lens). ELL-associated factor 2 (EAF2), a component of the ELL-mediated RNA polymerase II elongation factor complex, is a target gene and downstream factor of Wnt-4 signaling. Knockdown of Wnt-4 causes a failure of the development of the eye, whereas EAF2 can rescue the phenotype of loss of Wnt-4 function.^16,17^ The results of these studies suggested that EAF2 plays an important role in the development of the lenses. Deficiency of EAF2 causes an inhibition of eye development, particularly the lens development.^16,17^ UV radiation can stimulate the expression of EAF2.^15^ Melanin in the iris blocks the UV radiation through the iris. Therefore, dark pigmentation of the iris may slightly decrease the UV radiation pass through the iris and downregulates the expression of EAF2 slightly, which may results in a slight inhibition of development of lens and the slight decrease of the lens thickness. Third, the factors that modulate the iris color and LT may have some common gene polymorphisms, so that the LT co-relates with the iris color. These three hypotheses on the relationship between the color of the iris and the LT require further validation.

Some limitations should be acknowledged. First, the subjective grading protocol for iris color was subject to measurement bias. A more objective method for quantifying iris color may help to achieve more precise and reliable measurements. Nevertheless, the intraobserver agreement of the two grades was relatively good and the graders were masked to the subject's clinical characteristics while performing the grading. Thus, we believe the measurement bias might be minimal. Furthermore, our analysis was based on cross-sectional data and causal relationship cannot be determined. It is also likely that thicker lens may results in a lighter iris color, as discussed previously. In the end, Chinese had small variations in iris color, which is light brown to dark brown. Whether the findings observed in this study could be directly extrapolated to other ethnic groups who had a larger variations in iris color, such as the whites remains unclear.

In conclusion, our study suggested a possible connection between iris color and LT in Chinese teenagers. The association needs to be confirmed and replicated in other populations and mechanisms underlying the association warrant further clarification.

## References

[1] Nischler C, Michael R, Wintersteller C (2013). Iris color and visual functions. *Graefes Arch Clin Exp Ophthalmol* Jan.

[2] Cumming RG, Mitchell P, Lim R (2000). Iris color and cataract: the Blue Mountains Eye Study. *Am J Ophthalmol* Aug.

[3] Leske MC, Wu SY, Nemesure B, Hennis A (2002). Risk factors for incident nuclear opacities. *Ophthalmology*.

[4] (2001). Risk factors associated with age-related nuclear and cortical cataract: a case-control study in the Age-Related Eye Disease Study, AREDS Report No. 5. *Ophthalmology*.

[5] Fraser-Bell S, Choudhury F, Klein R, Azen S, Varma R; (2010). for the Los Angeles Latino Eye Study Group. Ocular risk factors for age-related macular degeneration: the Los Angeles Latino Eye Study. *Am J Ophthalmol*.

[6] Chaine G, Hullo A, Sahel J (1998). Case-control study of the risk factors for age related macular degeneration. France-DMLA Study Group. *Br J Ophthalmol*.

[7] Schmidt-Pokrzywniak A, Jockel KH, Bornfeld N, Sauerwein W, Stang A (2009). Positive interaction between light iris color and ultraviolet radiation in relation to the risk of uveal melanoma a case-control study. *Ophthalmology*.

[8] Pan CW, Qiu QX, Qian DJ (2018). Iris colour in relation to myopia among Chinese school-aged children. *Ophthalmic Physiol Opt* Jan.

[9] Ozeki H, Ito S, Wakamatsu K, Thody AJ (1996). Spectrophotometric characterization of eumelanin and pheomelanin in hair. *Pigment Cell Res* Oct.

[10] Pan CW, Wu RK, Liu H, Li J, Zhong H (2018). Types of lamp for homework and myopia among Chinese school-aged children. *Ophthalmic Epidemiol*.

[11] Pan CW, Wu RK, Wang P, Li J, Zhong H (2018]). Reduced vision, refractive errors and health-related quality of life among adolescents in rural China [published online March 25. *Clin Exp Optom*.

[12] National High Blood Pressure Education Program Working Group on High Blood Pressure in Children and Adolescents (2004). The fourth report on the diagnosis, evaluation, and treatment of high blood pressure in children and adolescents. *Pediatrics*.

[13] Hu DN, Simon JD, Sarna T (2008). Role of ocular melanin in ophthalmic physiology and pathology. *Photochem Photobiol*.

[14] Hu DN (2008). Methodology for evaluation of melanin content and production of pigment cells in vitro. *Photochem Photobiol*.

[15] Xiao F, Zhang JS, Zhao JY, Wu D (2012). Regulation of Eaf2 in mouse lens cells apoptosis induced by ultraviolet radiation. *Int J Ophthalmol*.

[16] Maurus D, Heligon C, Burger-Schwarzler A, Brandli AW, Kuhl M (2005). Noncanonical Wnt-4 signaling and EAF2 are required for eye development in Xenopus laevis. *EMBO J*.

[17] Li M, Wu XB, Zhuang FF, Jiang SY, Jiang MS, Liu YH (2003). Expression of murine ELL-associated factor 2 (Eaf2) is developmentally regulated. *Dev Dynam*.

